# Bait-attending amphipods of the Tonga Trench and depth-stratified population structure in the scavenging amphipod *Hirondellea dubia* Dahl, 1959

**DOI:** 10.7717/peerj.5994

**Published:** 2018-12-07

**Authors:** James P.A. Wilson, Kareen E. Schnabel, Ashley A. Rowden, Rachael A. Peart, Hiroshi Kitazato, Ken G. Ryan

**Affiliations:** 1 School of Biological Sciences, Victoria University of Wellington, Wellington, New Zealand; 2 Coasts & Oceans, National Institute of Water & Atmospheric Research Ltd., Wellington, New Zealand; 3 Institute of Biogeosciences, Japan Agency for Marine-Earth Science and Technology, Yokosuka, Kanagawa, Japan

**Keywords:** Amphipoda, Hadal, Assemblage composition, Deep sea, Lysianassoidea, Ontogenetic stratification, Zonation

## Abstract

**Background:**

The hadal zone encompasses the deepest parts of the world’s ocean trenches from depths of ∼6,000–11,000 m. The communities observed at these depths are dominated by scavenging amphipods that rapidly intercept and consume carrion as it falls to the deepest parts of the trenches. New samples collected in the Tonga Trench provide an opportunity to compare the amphipod assemblages and the population structure of a dominant species, *Hirondellea dubia* Dahl, 1959, between trenches and with earlier data presented for the Tonga Trench, and other trenches in the South Pacific.

**Methods:**

Over 3,600 individual scavenging amphipods across 10 species were collected in seven baited traps at two sites; in the Horizon Deep site, the deepest part of the Tonga Trench (10,800 m) and a site directly up-slope at the trench edge (6,250 m). The composition of the bait-attending amphipods is described and a morphometric analysis of *H. dubia* examines the bathymetric distribution of the different life stages encountered.

**Results:**

The amphipod assemblage was more diverse than previously reported, seven species were recorded for the first time from the Tonga Trench. The species diversity was highest at the shallower depth, with *H. dubia* the only species captured at the deepest site. At the same time, the abundance of amphipods collected at 10,800 m was around sevenfold higher than at the shallower site. *H. dubia* showed clear ontogenetic vertical structuring, with juveniles dominant at the shallow site and adults dominant at the deep site. The amphipods of the deeper site were always larger at comparable life stage.

**Discussion:**

The numbers of species encountered in the Tonga Trench is less than reported from the New Hebrides and Kermadec trenches, and six species encountered are shared across trenches. These findings support the previous suggestion that the fauna of the New Hebrides, Tonga and Kermadec Trenches may represent a single biogeographic province. The ontogenetic shift in *H. dubia* between the two Tonga Trench sites supports the hypothesis of interspecific competition at the shallower bathymetric range of the species, and the presence of competitive physiological advantages that allow the adults at the trench axis to exploit the more labile organic material that reaches the bottom of the trench.

## Introduction

The hadal zone extends from around 6,000 m depth to the deepest areas of the ocean (terminology according to Jamieson, 2015) and includes some of the most remote and unexplored environments on earth. Scavenging amphipods are a defining element of hadal communities, with the earliest records of this fauna provided by Dahl (1959), Wolff (1960), Hessler et al. (1978), and Ingram & Hessler (1983). Amphipods dominate the scavenging community at carrion falls, particularly at the greater depths of a trench, and are key prey items for predators of the hadal zone (Jamieson et al., 2009a). They have been recovered by almost every baited trap set at hadal depths (Hessler et al., 1978; Blankenship et al., 2006; Eustace et al., 2013; Lacey et al., 2016), and are frequently recorded by baited cameras and deep trawls (Wolff, 1960; Jamieson, Solan & Fujii, 2009b; Gallo et al., 2015). Jamieson (2015) states that the dominance of scavenging amphipods ‘at full ocean depth cannot be understated’ and provides multiple arguments why they might be particularly well-adapted to low-food environments. These adaptations allow them to rapidly detect, intercept, and consume carrion, and attend bait in very high abundance. In addition, amphipods can survive long periods of starvation and supplement their diet between carrion fall events (Jamieson, 2015). In the past, hadal amphipods were thought to rely solely on necrophagy for sustenance (De Broyer, Nyssen & Dauby, 2004), however, it is likely that they also undertake detritivory, carnivory, and cannibalism (Blankenship & Levin, 2007; Jamieson et al., 2010). This dietary diversity allows different amphipod species, by partitioning food sources, to co-exist at similar depths in the organic-matter limited trenches (Blankenship et al., 2006).

Since hadal depths are notoriously difficult to sample, there has so far only been limited opportunity to broadly compare the amphipod fauna across hadal regions. In the South–West Pacific Ocean, Blankenship et al. (2006) and Blankenship & Levin (2007) provided the first records for four amphipod species in the adjacent Tonga and Kermadec trenches. More recently, collections by HADal Environment and Education Program (HADEEP) have expanded sampling across trenches in the South–West and South–East Pacific, to provide further insight into the amphipod fauna (Jamieson, Solan & Fujii, 2009b; Jamieson et al., 2011; Fujii et al., 2013; Lacey et al., 2016, 2017). Collectively, these studies have allowed for both a detailed characterization of the amphipod community structure of the Kermadec, New Hebrides and Peru-Chile trenches, and a comparison with the nearby abyssal assemblages. Lacey et al. (2016) identified between seven (South Fiji Basin) and 21 (Kermadec Trench) species of amphipods across all depths and confirmed a clear distinction between abyssal and hadal communities. Some species appeared restricted to a single trench, yet the most dominant hadal species were documented at both the Kermadec and New Hebrides trenches, with different communities documented at the Peru-Chile Trench. As a result, Lacey et al. (2016) suggested combining the current designation of two hadal biogeographic provinces (Belyaev, 1989) into one province for hadal environments in the South West Pacific Ocean.

Amphipod assemblages within trench environments appear to be vertically stratified with species confined to a relatively narrow bathymetric range within each trench. The vertical stratification of species has been related to physiological tolerances and metabolic processes that are in part the result of temperature and pressure gradients (Brown & Thatje, 2014, 2018; Smith, Brown & Thatje, 2015). Interestingly, the observed depth partitioning between two dominant species, *Hirondellea dubia* Dahl, 1959 and *Bathycallisoma schellenbergi* (Birstein & Vinogradov, 1958), in the Tonga, Kermadec, and New Hebrides trenches varied between trenches, although an overall pattern of dominance at the intermediate (*B. schellenbergi*) and deepest depths (*H. dubia*) remained (Blankenship et al., 2006; Lacey et al., 2017). Thus, the vertical distribution of species is not likely to be solely driven by pressure and temperature but is also a result of other environmental factors and/or competition/predation.

The genus *Hirondellea* is found throughout the world’s ocean trenches, and often dominates assemblages at the deepest depths of trenches (Hessler et al., 1978; Blankenship et al., 2006; Eustace et al., 2013; Horton & Thurston, 2013; Kilgallen, 2014; Lacey et al., 2017). The species *H. dubia* is distributed across trenches in the South West Pacific Ocean, including the Kermadec, Tonga and New Hebrides trenches (Dahl, 1959; Blankenship et al., 2006; Ritchie, Jamieson & Piertney, 2015; Lacey et al., 2016, 2017), and has been found at abyssal depths near the Mariana Trench (Ritchie, Jamieson & Piertney, 2015). *H. dubia* dominates the deepest depths (>7,500–8,000 m) in the Kermadec and Tonga trenches, and occurs at baited traps in high abundances where no other species have been recorded to date (Blankenship et al., 2006; Lacey et al., 2017). Blankenship et al. (2006) report up to 17,800 amphipods in a single trap deployed at just over 8,700 m in the Tonga Trench, and for the first time reported an ontogenetic structuring of the population with depth. This pattern was subsequently also shown for *H. gigas* in the Izu-Bonin Trench (Eustace et al., 2013), and *B. schellenbergi* in the Kermadec and New Hebrides trenches (Lacey et al., 2017).

The deployment of seven baited traps at two sites in the Tonga Trench during the Japanese 2013 Quest for the Limit of Life (QUELLE) expedition provided an opportunity to expand on previous reports of amphipod assemblages in the South West Pacific Ocean, and compare the new findings in the context of the recently presented data from the Kermadec and New Hebrides trenches (Lacey et al., 2016, 2017; Ritchie, Jamieson & Piertney, 2017), as well as previous data on the vertical distribution of *H. dubia* from the Tonga Trench (Blankenship et al., 2006).

## Materials and Methods

### Study sites and sample collection

**Figure 1:**
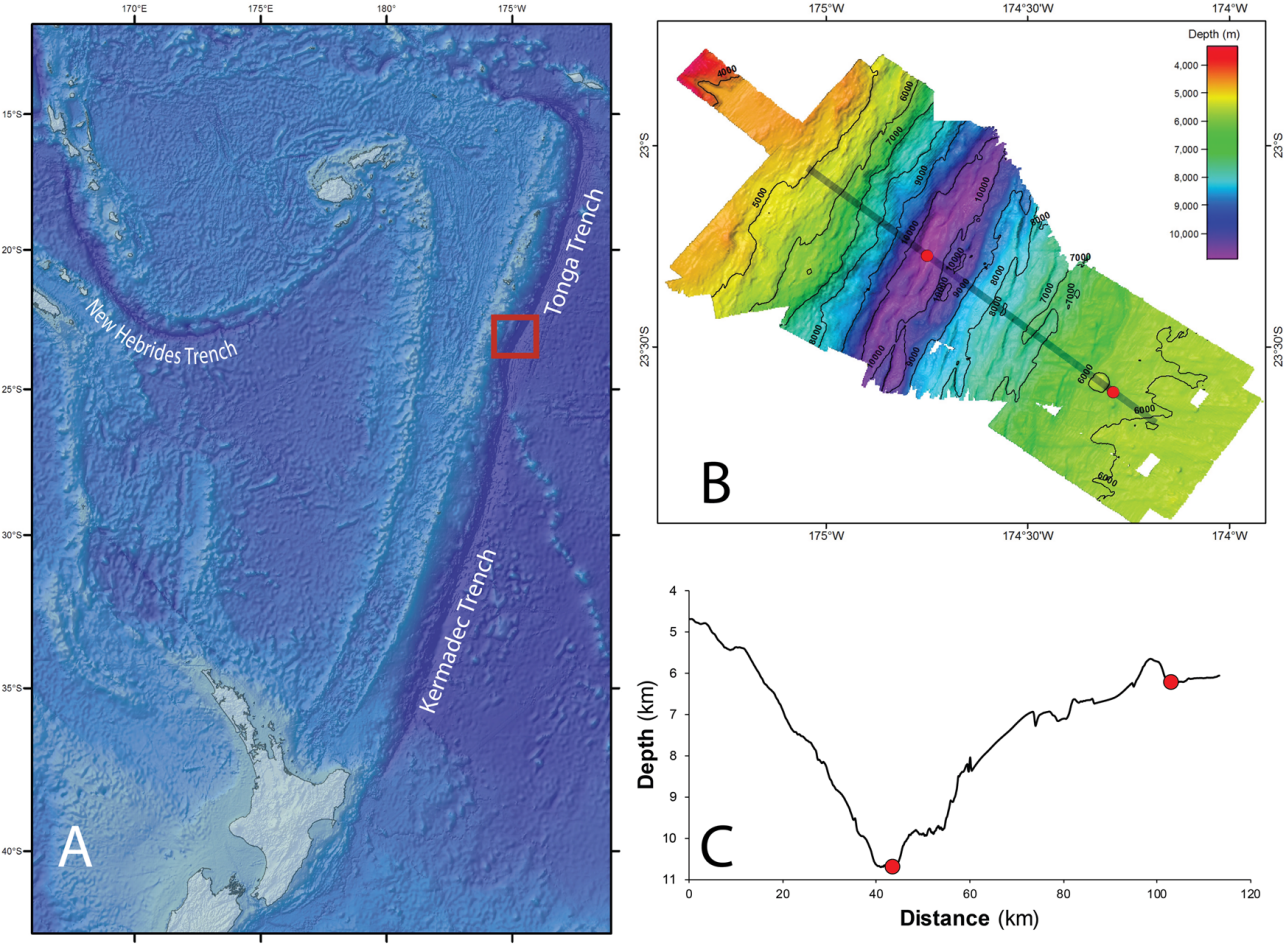
Tonga Trench Study site in the Southwest Pacific Ocean. (A) The red square identifies the study site where samples were collected. Bathymetry (B) and bathymetric profile (C) of the area studied (Red dots indicate the two sampling sites, ∼10,800 m in the trench axis, and ∼6,250 m on the trench outer slope. Figure adapted from Leduc et al. (2016) with permission (© Elsevier).

The Tonga Trench is located in the South West Pacific Ocean, and comprises the northern portion (from ∼15°S to ∼25°S) of the trench system that runs between Samoa and Tonga in the north and New Zealand in the south (Wright et al., 2000). Its deepest point is 10,882 m, a site known as the Horizon Deep, and it is separated from the neighboring Kermadec Trench in the south by the Tonga Platform, a sill that rises to approximately 5.5 km in depth (Fig. 1A).

With permission from the Ministry of Foreign Affairs and Trade of the Government of the Kingdom of Tonga, the Tonga Trench was visited in 2013 as part of the QUELLE round-the-world expedition conducted by the Japan Agency for Marine-Earth Science and Technology using the submersible *Shinkai 6500* and its support vessel *Yokosuka*. Samples of scavenging amphipods were collected using baited traps from two depths within the trench: at approximately 10,800 m at the Horizon Deep, and directly up-slope at approximately 6,250 m on the edge of the trench. These sites were approximately 60 km apart (Figs. 1B and 1C). Baited traps (25 × 25 × 36 cm with two six cm diameter openings and three mm mesh) were deployed on benthic landers at the Horizon Deep site on four occasions, and on three occasions by a submersible at the trench edge site. The bait (raw fish) was placed in fine mesh bags (mesh size approx. two mm) attached to the inside wall of the trap. The heights of traps from the seafloor varied between the sampling occasions (0, 1.5, and 1.8 m). Details on location, specific depth, and deployment date and time are listed in Table 1.

**Table 1:** Sample locations.

	Site/Dive No.	Depth (m)	Latitude	Longitude	Date	Gear	Time at sea floor hours:minutes
1	1370/1–5	6,255	23:36.7500:S	174:17.3444:W	October 15, 2013	Trap deployed by submersible and suction sampler; trap height zero m.	3:00
2	1–1	6,256	23:36.6771:S	174:16.8787:W	October 9, 2013	Sediment profile lander; two traps at 1.8 m above seafloor	2:46
3	1–2	6,253	23:36.6344:S	174:16.8133:W	October 10, 2013	Sediment profile lander; two traps at 1.8 m above seafloor	11:16
4	2–1	10,817	23:16.4294:S	174:44.9826:W	October 11, 2013	Camera-corer lander; two traps at 1.5 m above seafloor and one at zero m	15:16
5	2–2	10,807	23:16.5298:S	174:44.8380:W	October 12, 2013	Sediment profile lander; two traps at 1.8 m above seafloor	7:02
6	2–3	10,807	23:16.5085:S	174:45.1347:W	October 13, 2013	Camera-corer lander; two traps at 1.5 m above seafloor and one at zero m	8:06
7	2–4	10,805	23:16.54365:S	174:45.2294:W	October 14, 2013	Sediment profile lander; two traps at 1.8 m above seafloor	9:43

**Notes:**

Sample details for scavenging amphipods, Site 1 corresponds to trench edge site, site 2 corresponds to Horizon Deep site. Time at sea floor indicate the hours and minutes that each sample had to collect scavengers from the sea floor.

Environmental data for the lander deployments was collected with a conductivity, temperature and depth instrument (SBE49, Sea-Bird Electronics, Bellevue, WA, USA) and a custom-built microprofiling system containing O_2_ microelectrodes. Sediment characteristics were obtained from sediment cores and subsequently analysed for sediment-bound chlorophyll *a* (Chl *a*), organic carbon content and phytopigments (see Wenzhöfer et al., 2016 for details) (Table 2).

**Table 2:** Environmental characteristics.

Site	Temperature (°C)	O_2_ uptake (μmol^−2^ d^−1^)	Chl a* (mg m^−2^)	Phaeophytin* (mg m^−2^)	Prokaryotic abundance* (cells cm^−2^)	^210^Pb_ex_ inventory* (kBq m^−2^)
Abyssal (6,250 m)	1.2	92 ± 44 (*n* = 16)	4.5 ± 0.3 (*n* = 2)	21 ± 3.7 (*n* = 2)	7.2 × 10^7^ ± 0.13 × 10^7^	9.5 ± 4.6
Hadal (10,800 m)	2	225 ± 50 (*n* = 7)	29 ± 1.8 (*n* = 2)	125 ± 43.8 (*n* = 2)	12 × 10^7^ ± 0.14 × 10^7^	207.6 ± 3.6

**Notes:**

Benthic fluxes and depth-integrated sediment parameter from hadal and abyssal trench sites (Wenzhöfer et al., 2016).

* Depth-integrated values from retrieved sediment cores (0–15 cm).

### Sample processing

Amphipods sampled from each trap deployment were processed separately (Table 1) and were initially fixed in 100% ethanol and later transferred to 70–80% ethanol for species identification, counting, and measuring. A small number of specimens were randomly removed from the most abundant samples (i.e. those that were later subsampled, see below) for separate analysis not reported upon here. Amphipods were identified to the lowest possible taxonomic level following D’Udekem D’Acoz & Havermans (2015), Barnard & Ingram (1990), Dahl (1959), Ingram & Hessler (1987), Barnard & Karaman (1991), Jamieson et al. (2013), Kilgallen (2014), Kilgallen & Lowry (2015), Shulenberger & Barnard (1976), Birstein & Vinogradov (1955, 1958) and Barnard & Shulenberger (1976) using a stereoscopic microscope (LEICA MZ 12.5; Leica Camera AG, Germany) and a compound microscope (Zeiss Axioskop 2plus; Carl Zeiss AG, Germany). The specimens are deposited at the NIWA Invertebrate Collection in Wellington, New Zealand.

*Hirondellea dubia* individuals were counted, sexed, imaged, and analysed morphometrically (see details below). After assessing the sample sizes and considering time constraints, a random subset of 450 individuals was processed when the numbers of individuals in a sample exceeded 450. In samples that contained fewer than 450 individuals, all the individuals were processed.

All morphometric analyses were conducted using the image analysis software ImageJ 1.49 (Abramoff, Magalhaes & Ram, 2004). Previously, the diameter of the 4th coxa has been used as a proxy for total length (Blankenship et al., 2006; Lacey et al., 2017), but, for example, Eustace et al. (2013) used the total body length for their comparisons of *H. gigas* populations. Here, the 4th coxa was often damaged and difficult to photograph and the total length was measured directly instead. The total length is measured along the dorsal midline, from the tip of the rostrum to the anterior end of the telson, separately recording lengths for the head, pereonite 1, pereonite 2–7, the pleosome, and the urosome. Measurements were then combined to create a total length value. Each measurement was conducted using the Segmented Line Tool in ImageJ. Typically, this measurement composed of a single line from the start of the segment to the end, however, for larger segments the line was broken up into a series of connected lines (sensu Chapelle, 1995).

The curvature of the dorsal aspect varied among individuals and could not be corrected in the preserved amphipods without causing damage. In order to capture any variation in total length measurements caused by dorsal curvature, four categories ranging from 1 to 4 were recorded. These categories were assigned to amphipods based on an angle created by two lines from the tip of the rostrum to the anterior margin of the 5th pereonite and to the base of the telson. Curvature angles of 200–160° (most straight), 160–120°, 120–90°, and <90° (most curved) were rated 1, 2, 3, and 4, respectively.

All the specimens were sexed and separated into seven life stage categories based on criteria adapted from Hessler et al. (1978) (Table 3). The number associated with each life stage represents the stage of development and is not representative of age and cannot be compared across sexes (i.e. male 3 is the fully mature male, while female 3 is still immature). Juveniles were classified when an individual was too small to be sexed or when genitalia were indistinguishable.

**Table 3:** Life stages of *H dubia*.

Sex/life stage	Life stage code	Description
Juvenile	J	No visible papillae or oöstegites
Male 2	M2	Penile papillae present, calceoli absent from antenna
Male 3	M3	Penile papillae present, calceoli present, slightly elongated second antenna
Female 2	F2	Short oöstegites trace can be found on pereiopods 3–5
Female 3	F3	Small oöstegites protrude from pereiopods 3–5
Female 3a	F3a	Oöstegites protrude out over the abdomen
Female 4	F4	Large oöstegites possessing setae

**Note:**

Criteria for assigning life stage to *H. dubia* individuals (adapted from Hessler et al., 1978).

### Statistical analyses

Analyses of morphometric and demography data were conducted using the IBM SPSS statistics software (version 22.0, 2015; Armonk, NY, USA) and R (version 3.4.1; R Core Team, 2017). Data were analysed for normality and homogeneity for the tests that require these attributes. In most cases, assumptions of normality and homogeneity were met. In some cases, normality was only approaching significance, however, after evaluating the normal Q–Q plots these cases were considered acceptable for parametric analysis.

A Pearson’s Chi-square test of contingencies was used to evaluate sex composition of the sample populations. Female and male life stages were each pooled into two groups, and sexual composition (as a percentage of the total population) was then compared between the samples from depths of 6,250 and 10,800 m. One-way between groups ANOVA was used to assess the impact of body curvature and trap height on total length, and both were included as a covariate in the subsequent analyses. Factorial between-groups ANCOVA was used to analyse *H. dubia* size structure between life stages and between sites at 6,250 m (trench edge) and 10,800 m (Horizon Deep) depths. A simple effects analysis was then performed by running an ANOVA with split file groups based on life stage. The ANOVA assessed how depth impacted the variability of total length for each life stage. The significance of the results from this test were interpreted at *p* < 0.001 to control for the inflation of family-wise error rates that occurs when conducting multiple comparisons on the same set of data.

Non-parametric Kendall’s rank correlation was used to examine the effect of deployment time on abundance and size of amphipods.

## Results

### Species composition

The baited traps successfully captured more than 3,600 individual scavenging amphipods at two depths in the Tonga Trench (the only non-amphipod organisms collected were two mysids at the shallower site, and these are not here considered further). A total of 449 individual amphipods were captured at the trench edge (6,250 m) and 3,175 were collected at the Horizon Deep site (10,800 m) (Table 4).

**Table 4:** Amphipod species composition of the Tonga Trench.

Family	Species	Regions (* new record for Tonga Trench)			Stations	Depth	Count (site 1)	Count (site 2)
Alicellidae	*Alicella gigantea*	TT*	KT	NHT	2, 3	6,253–6,256	2	
	*Paralicella tenuipes*	TT*	KT	NHT	2	6,256	13	
	*Paralicella* cf. *caperesca*	TT*	KT	NHT	2	6,256	75	
	*Paralicella* cf. *fusiformis*	TT*			2, 3	6,253–6,256	17	
Cyclocaridae	*Cyclocaris sp.* (cf *tahitensis*)*	TT*	KT?		2, 3	6,253–6,256	25	
Eurytheneidae	*Eurythenes gryllus/sigmiferus*	TT	KT?	NHT?	1–3	6,253–6,256	137	
Hirondelleidae	*Hirondellea dubia*	TT	KT	NHT	2–7	6,253–10,807	72	3,175
	*Hirondellea* sp.*	TT*			2	6,256	13	
Scopelocheiridae	*Bathycallisoma schellenbergi*	TT	KT	NHT	2, 3	6,253–6,256	83	
Uristidae	*Abyssorchomene distinctus*	TT*	KT		2, 3	6,253–6,256	12	

**Notes:**

Composition and abundance of bait-attending amphipods recovered from seven stations sampled across depths between 6,253 m (site 1) and 10,807 m (site 2) in the Tonga Trench (TT). Known species records in the Kermadec Trench (KT) and New Hebrides Trench (NHT) are included (Lacey et al., 2016).

* Indicates potentially new species. Station numbers refer to Table 1.

Amphipod assemblage composition varied substantially between the two sampling sites. A total of 10 amphipod species were sampled, but only *H. dubia* Dahl, 1959 was found at both sites. All remaining species were restricted to the shallower site (Table 4). Two species are possibly new to science: *Hirondellea* sp. (Hirondelleidae) and a *Cyclocaris* cf. *tahitensis* Stebbing, 1888 (Cyclocaridae) and await formal description (R. Peart et al., 2018, unpublished data). Four species of Alicellidae, *Alicella gigantea* Chevreux, 1899, *Paralicella* cf. *caperesca* Shulenberger & Barnard, 1976, *P. tenuipes* Chevreux, 1908 and *P.* cf. *fusiformis* (Birstein & Vinogradov, 1955), and the uristid species *Abyssorchomene distinctus* (Birstein & Vinogradov, 1960), are reported for the first time from the Tonga Trench (Table 4). The only species of *Eurythenes* (Eurytheneidae) encountered among the samples was *Eurythenes sigmiferus*, recently described by D’Udekem D’Acoz & Havermans (2015).

The trench edge site assemblage was dominated by *E. sigmiferus* with 137 individuals (∼30%), and with progressively diminishing numbers of *B. schellenbergi*, *P.* cf. *caperesca*, and *H. dubia* between 83 and 72 (18–16%) in the overall sample (Table 4, site 1). However, 3,175 specimens of *H. dubia* were collected at the Horizon Deep site, a number nearly sixfold higher than the total number of specimens collected at the trench edge site (Table 4, site 2).

### Selection of covariates

The size and abundance of *H. dubia* varied significantly between trap heights (*F*_2.1130_ = 39.487, *p* < 0.001 and *F*_2.10_ = 6.966, *p* < 0.018, respectively). Traps set at the sea floor captured at least three times the number of, significantly larger, *H. dubia* specimens than those set at 1.5 or 1.8 m from the seafloor. Based on these results, the effects of trap height were included as a covariate in the factorial between-groups ANCOVA analysis.

Amphipods with higher curvature ratings had significantly higher total lengths (*F*_3.1173_ = 46.563, *p* < 0.001). Therefore, the total body length was included for all animals (see Materials and Methods above) with curvature as a covariate in the factorial between-groups ANCOVA analysis of *H. dubia* size structure described below.

The effect of the deployment time (range ∼3–15 h, Table 1) was assessed for the deep deployments only since the numbers of specimens collected at the shallow site were insufficient. A non-parametric Kendall’s rank correlation provided mixed results. For all amphipods, the correlation was insignificant (*p* = 0.21) with a weakly positive relationship between deployment time and numbers of specimens (τ = 0.029). When considering the separate stages, some relationships were significant with positive trends (*p*-values F2 and F3a, *p* < 0.001, M2, *p* = 0.004) and other stages were not significant (F3, F4, and M3 *p* > 0.1). Therefore, there was a weak indication that amphipod size increases for some stages as deployment times increase, but since the overall relationship was not significant, deployment time was not included as a covariate in the final analysis.

### Ontogenetic structure and body size of *H.* *dubia*

Of the 3,247 *H. dubia* collected from both depths, 1,471 individuals were sexed, imaged, and then morphometrically analysed. Of the analysed individuals, 72 were collected from the 6,250 m, and 1,399 from 10,800 m depth. No brooding females or intersex amphipods were captured (Table 5).

**Table 5:** Number and length measurements of *H. dubia* for two sites in Tonga Trench.

Depth	Length (mm)	F4	F3a	F3	F2	J	M2	M3	Total (n)
6,250 m	*n*	**0**	**2**	**0**	**4**	**65**	**1**	**0**	**72**
	Min	na	14.6	na	10.6	4.8	11.1	na	
	Mean		15.1		11.3	6.2			
	Max		15.7		12.2	7.8			
10,800 m	*n*	**40**	**140**	**288**	**420**	**18**	**383**	**110**	**1,399**
	Min	15	12.1	11.7	8.7	9.1	9.2	11.7	
	Mean	18.3	17.2	16.1	13.8	10.1	14.3	16.5	
	Max	22	22.4	22.4	19	11.1	21.7	19.9	
Total (*n*)		**40**	**142**	**288**	**424**	**83**	**384**	**110**	**1,471**

**Note:**

Number of individuals (*n*, in bold) and total body length (mm) of *H. dubia* by sex and life stages across two depths ∼6,250 and ∼10,800 m.

**Figure 2:**
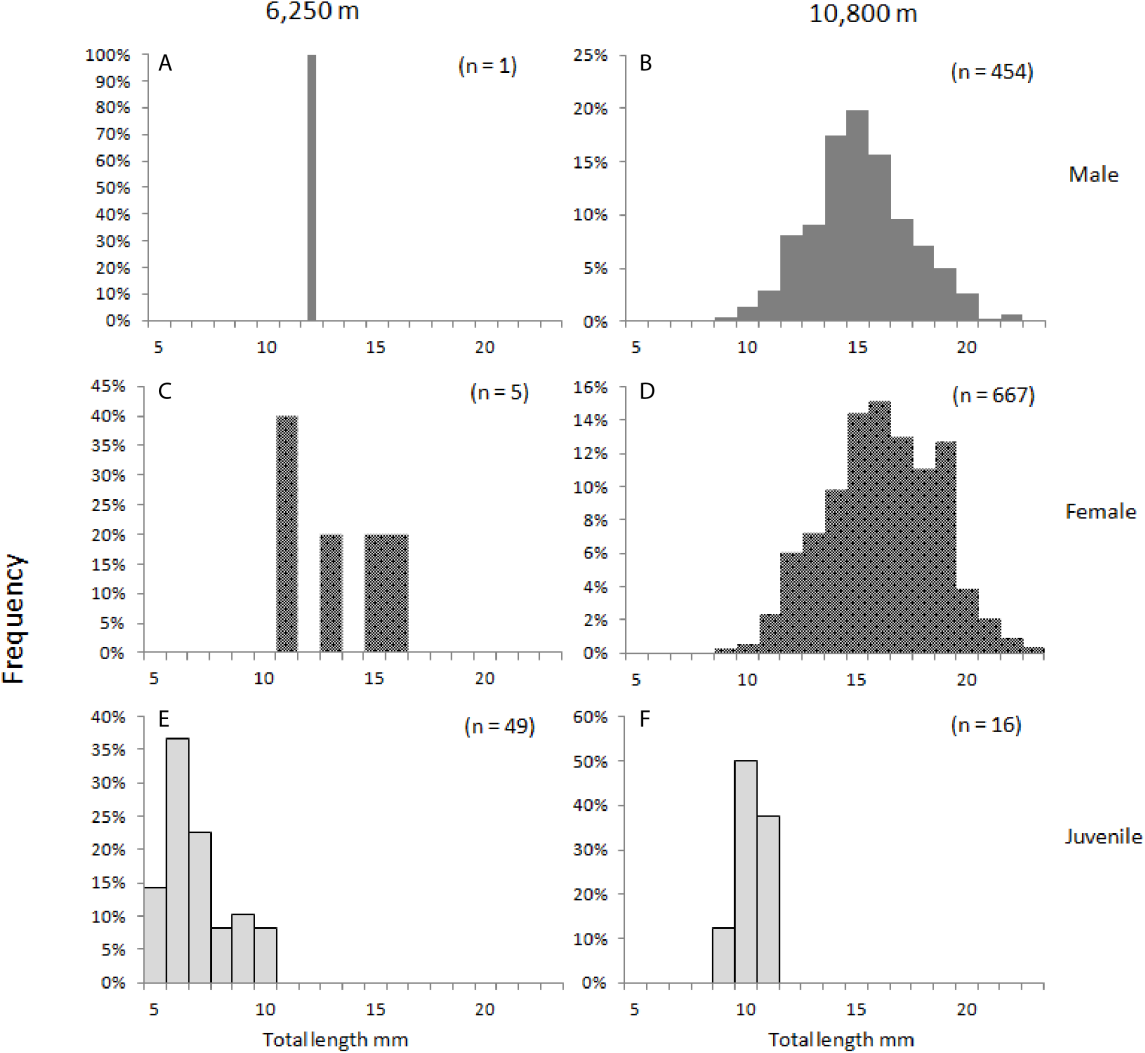
*Hirondellea dubia* frequency distribution plot. Frequency distribution plot of *H. dubia* size structure for the two sampling depths, shallow (A, C, E) and deep (B, D, F), and across sexes, male (A, B), female (C, D) and juvenile (E, F). Male (M2, M3) and Female (F2, F3, F3a, F4) life stages were grouped into respective sexes.

In 285 specimens, the thorax or abdomen was substantially ruptured or distorted or the urosome was missing, hence, the total length could not be determined, and they were excluded from the morphometric analysis. For the 1,186 specimens of *H. dubia* measured, the total length ranged from 4.8 mm for the smallest juvenile, to maximum total lengths of 21.7 mm for males and 22.4 mm for females (Table 5; Fig. 2). The smallest specimens were collected at the trench edge site (range of total length: 4.8–15.7 mm), and the largest animals were collected at the trench axis (range of total length: 8.7–22.4 mm). The difference in mean total lengths of *H. dubia* at 10,800 m depth ($\bar x = 15.26\,{\rm{mm}}$) was statistically significant compared to that at 6,250 m ($\bar x = 6.98\,{\rm{mm}}$), (*F*_1.1164_ = 16.2, *p* < 0.001) (Fig. 2). Mean total length also varied significantly among life stages, between 6.2 and 15.1 mm for the shallower site, and 10.1–18.3 mm for the deep site (*F*_6.1164_ = 80.549, *p* < 0.001).

Juveniles made up 90% (65 individuals) of the population at 6,250 m compared to 1% (18 individuals) at 10,800 m (J in Table 5). At both depths, females were more abundant than males. At the trench edge site, only six females and one male were sampled, and no fully mature life stages (M3 and F4) were recorded, while at the Horizon Deep site, 63% of the samples were females (888 individuals) and 36% were males (493 individual). Generally, the smaller life stages (F2 females and M2 males, and juveniles J) dominated at both depths, to a combined 97% at the 6,250 m and 59% at the 10,800 m depth site. Conversely, only two larger females (F3a) were collected at the shallower site compared to a combined 578 individuals, or 41%, of larger adults (F3–F4 and M3) at the deep site (Table 5). Overall, the sex composition was significantly different between depths, (χ^2^_2.200_ = 1,018.7, *p* < 0.001), with 80% of the variability in composition being described by depth (Cramer’s Ѵ = 0.82).

**Figure 3:**
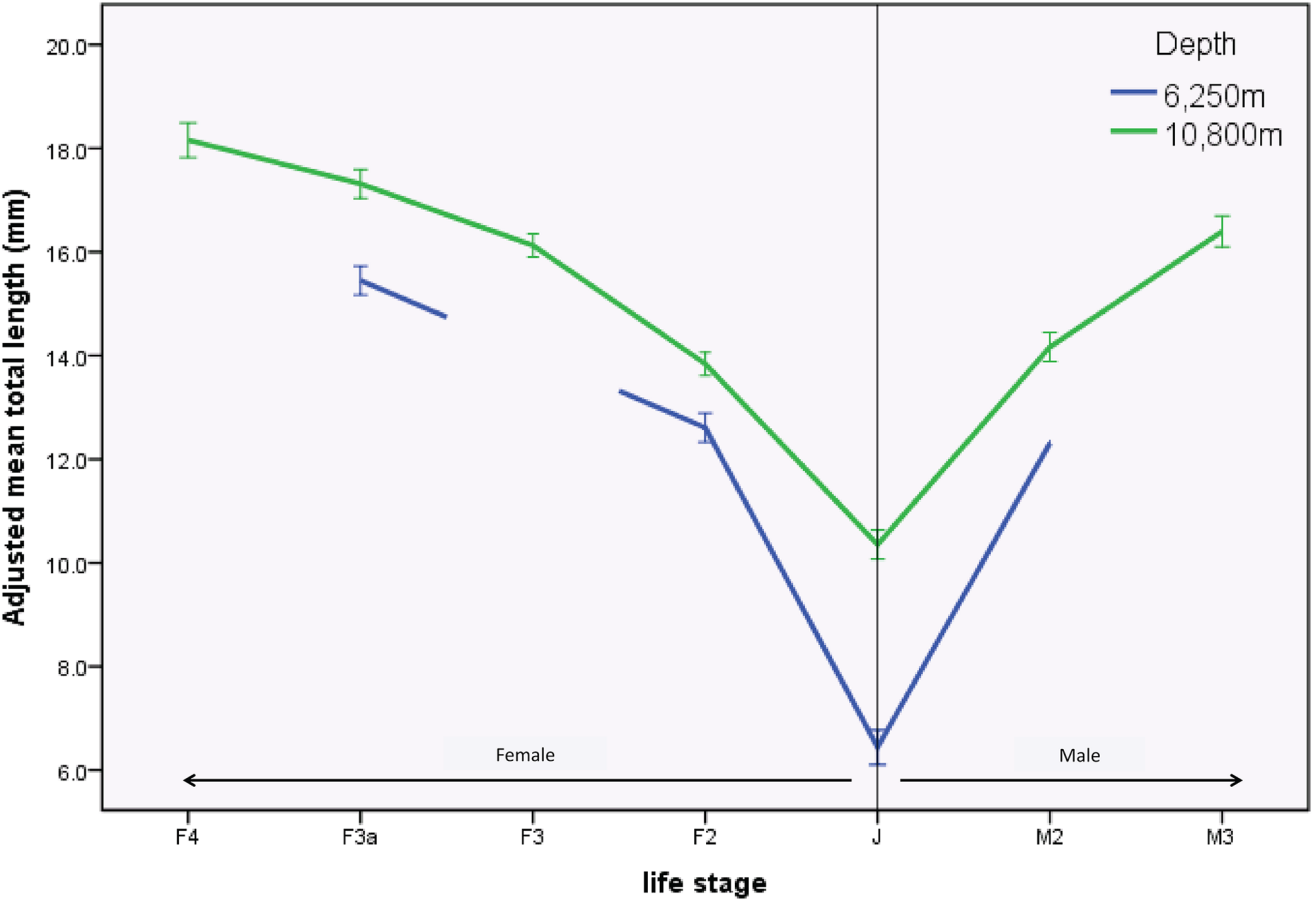
Profile plot of *H. dubia* size structure at two depths in the Tonga Trench. *Hirondellea dubia* size structure for the two sampling depths and across life stages. The adjusted mean total length on the *y* axis is an estimated value based on the influence of covariates. Covariates appearing in the graph are evaluated at the following values: Curve rating (1–4) = 2.591, trap height (m) = 0.850. Means of life stages absent from the 6,250 m site were *n* on-estimable and were not plotted. Error bars are standard error.

There were significant differences in sizes between depths as shown in the length-frequency distributions (Fig. 2). The difference in mean size between depths was significantly greater in juveniles (J) and M2 than in the other life stages (*F*_3.1164_ = 2.968, *p* = 0.03). This result was further examined by a simple effects analysis. The combination of depth and life stage significantly influenced the between-depth difference between some of the life stages; J (*F*_1.1164_ = 70.155, *p* < 0.01), and M2 (*F*_1.1164_ = 6.776, *p* < 0.01) were significantly smaller at the 6,250 m site compared to the 10,800 m site, relative to differences between depths seen in life stages F2 and F3a (Table 3; Fig. 3).

## Discussion

### Tonga Trench amphipod assemblage

Little is known about the scavenging amphipod assemblage of the Tonga Trench. Data presented for four species collected from three sites (10 stations) between 6,252 and 10,787 m by Blankenship et al. (2006) and Blankenship & Levin (2007) remain the only previous records for the region. Here, we report on 10 species collected at two sites (seven stations) and at a similar depth range of 6,253–10,807 m. The Horizon Deep site is in very close proximity (∼ five km) to three stations sampled previously (F/V 2, 10, and 11) and the trench edge site lies at the same depth and about 28 km north–east of F/V 4 (see Blankenship et al., 2006). We report seven species for the first time from the Tonga Trench, two of which might be new to science (*P.* cf. *fusiformis* and *Hirondellea* sp.; R. Peart et al., 2018, unpublished data). Previously reported were both *B. schellenbergi* (Birstein & Vinogradov, 1958) and *H. dubia* Dahl, 1959 (Blankenship et al., 2006; Blankenship & Levin, 2007). A third species, *Eurythenes gryllus* (Lichtenstein, 1822), was also reported previously by Blankenship et al. (2006). Until recently, *E. gryllus* was considered a cosmopolitan species, however, D’Udekem D’Acoz & Havermans (2015) identified nine distinct genetic lineages and described five new species and, subsequently, Narahara-Nakano, Nakano & Tomikawa (2017) added another species from the Japanese Seas and discussed the deep-sea species diversity of the Pacific Ocean. The new species include *E. sigmiferus* that was also reported from the Kermadec Trench based on an image record (N. Kilgallen, 2017, personal communication), and which is the only species of *Eurythenes* encountered in this study. *E. gryllus* s.s. retains a broad bipolar distribution but with the deepest record known at 3,803 m (D’Udekem D’Acoz & Havermans, 2015) previous reports of *E. gryllus* from much deeper in the Tonga Trench are called into question. We consider the records provided for *E. gryllus* in the South West Pacific trenches by Blankenship et al. (2006) and Lacey et al. (2016) as doubtful and specimens need to be reexamined. Finally, Blankenship et al. (2006) also report an *Uristes* sp. nov. at depths between those sampled here (7,349–9,273 m). Subsequently, this species was resolved as belonging to a different family and assigned to the genus *Hirondellea* by Ritchie, Jamieson & Piertney (2017) using DNA sequencing. However, a member of the same family Uristidae, *Abyssorchomene distinctus* (Birstein & Vinogradov, 1960), was collected, a first record for the Tonga Trench, but previously recorded from the Kermadec Trench by Lacey et al. (2016).

Our data support previous findings that scavenging amphipod species diversity declines from the top of the trench to the bottom, with only one species (*H. dubia*) being present at the trench axis. Notably, the numbers of specimens collected varied, despite deployment times being similar (∼10 h). For example, 137 specimens of *E. sigmiferus* were collected in the present study, compared to 10 previously at a nearby site (Blankenship et al., 2006); 73 specimens of *H. dubia* were collected at 6,250 m, and at 10,800 m 3,174 specimens were captured, while Blankenship et al. (2006) did not encounter *H. dubia* shallower than a depth of 7,349 m and then report numbers of up to 17,800 specimens for a single station at 8,723 m and 884 specimens at the deepest of their stations (around 10,780 m). Hence, we extend the depth range known for this species in the Tonga Trench and observed greater abundances at the trench axis. However, comparisons should only be considered qualitative as the trap configurations were different. This remarkable depth range for *H. dubia* still falls within the known range observed for this species in the Kermadec Trench, where it is encountered as shallow as 4,700 m outside of the trench to its axis at 9,908 m (Lacey et al., 2016).

Lacey et al. (2017) provide the relative catch proportions of *B. schellenbergi* and *H. dubia* with depth for both the Tonga Trench and the Kermadec Trench, noting that *H. dubia* is the only species found at the deepest depths of the trench, while the reverse applies at intermediate depths in both trenches. Between ∼5,000 and 7,000 m in the Tonga Trench and between ∼7,000 and 8,000 m in the Kermadec Trench, *B. schellenbergi* dominates to the apparent exclusion of *H. dubia (*Lacey et al., 2017). Comparing the relative catch proportions for the two species in this study indicate that there is an overlap in the pattern observed by Lacey et al. (2017). At the trench axis our data agrees, with *H. dubia* present (1.00) and *B. schellenbergi* absent (0.00), however, our records from the 6,250 m site provide a different pattern with both amphipod species at low proportions (0.16 and 0.18 for *H. dubia* and *B. schellenbergi,* respectively). For the same depth, Lacey et al. (2017) refer to data provided by Blankenship et al. (2006) which show a dominant *B. schellenbergi* (0.72) and an absent *H. dubia* (0.00) instead. However, those data included in the figure of Lacey et al. (2017) for that depth were generated from a total of 32 amphipod specimens reported by Blankenship et al. (2006), 23 of which were *B. schellenbergi* (or 0.72 of the sample), with the other nine specimens being *E. gryllus* (or 0.28 of the sample). In our study, the *Eurythenes* species comprised a much larger proportion (137 specimens or 0.31 of the sample) of the total assemblage. In addition, seven other species were also recorded, and add to what we now know of the Tonga Trench assemblage. It is clear that the composition and depth-distribution of the amphipod assemblage is more complex than previously presented.

An interesting insight into the factors that might influence the distribution of the species is presented by the comparison of amphipod catch in traps set at different heights from the bottom. Blankenship et al. (2006) noted that two traps deployed in the Horizon Deep at 2.0 m above the bottom, instead of the typical 1.0 m, did not recover any animals. Our study recovered *H. dubia* in traps set at either 1.5 and 1.8 m above the sea floor, but traps set directly on the sea floor captured at least three times the number of *H. dubia* than those set above the seafloor, and the average amphipod size was significantly larger at the zero m traps. One explanation for this pattern may be the partitioning of foraging strategies, where larger (older) individuals outcompete smaller individuals at carrion falls on the sea floor. Another hadal amphipod, *E. gryllus*, displays partitioned vertical distribution, with individuals moving upward in the water column with increasing body size, thereby transitioning to a more pelagic lifestyle (Ingram & Hessler, 1987; Christiansen, Pfannkuche & Thiel, 1990; Jamieson et al., 2011). The mechanisms that attract high numbers of amphipods to bait appear to be highly specific and still very much unknown, as amphipods may swarm around particular pieces of bait while almost ignoring adjacent bait (Hessler et al., 1978). Previous samplings have been taken at a single trap height off the sea floor, or combined data across a variety of trap heights to represent a single sample (Ingram & Hessler, 1987; Fujii et al., 2013), and may have overlooked the influence of trap height. Future studies would benefit from analyzing how these attraction mechanisms contribute to the stratification of amphipod sizes in differing trap heights.

### Intertrench comparison of amphipod assemblages

Expanding on previous HADEEP project data published by Jamieson et al. (2011) and Fujii et al. (2013), Lacey et al. (2016) present a combined analysis of the bait-attending amphipod fauna from 21 bathyal to hadal sampling stations (1,488–9,908 m) from the Kermadec, Peru-Chile, and New Hebrides trench areas.

Close faunistic links with the Tonga Trench and other South West Pacific trenches are apparent; three species already recorded by Blankenship et al. (2006) and Blankenship & Levin (2007), and three of the seven species newly reported here for the Tonga Trench are shared with both the Kermadec and the New Hebrides trenches (Lacey et al., 2016). However, the full extent of this assessment is pending confirmation that all the material previously reported as *E. gryllus* belongs to *E. sigmiferus* and whether specimens identified as *P.* cf. *caperesca* are the same as the species referred to as *P. caperesca* in Lacey et al. (2016). In addition, both *P. caperesca* and *P. tenuipes* await a formal review following the detailed phylogeographic analysis by Ritchie, Jamieson & Piertney (2017). The uristid *Abyssorchomene distinctus* found in the Tonga Trench is also present in the Kermadec Trench (and the Peru-Chile Trench and South Fiji Basin) but so far unrecorded in the New Hebrides Trench (Lacey et al., 2016). And the specimens identified as *Cyclocaris* cf. *tahitensis* are sufficiently different that they are likely a new species which need to be compared with the *C. tahitensis* specimens collected from the Kermadec Trench. *P. fusiformis* is so far known from the northern Pacific and a similar species is reported here, although the specimens are sufficiently different that it is likely a new species, not recorded in any other South West Pacific trench. The same applies for *Hirondellea* sp. which does not appear to match any of the species described to date. The distribution of the *Hirondellea* species complex has so far been used to highlight regional hadal trench endemism, with *H. dubia* supposedly restricted to the South West Pacific trenches, *H. gigas* to the North West Pacific trenches, and three other species of *Hirondellea* restricted to the trenches of the South East Pacific Ocean (Kilgallen, 2014; Jamieson, 2015; Ritchie, Jamieson & Piertney, 2017).

Notably, species that are absent in the samples examined here from the Tonga Trench, but that have been reported in the region at comparable depths, might be expected to be collected here in the future. These are two species of each of the genera *Orchomenella*, *Paracallisoma*¸ *Tryphosella*, and *Valettietta* as well as two other species of *Abyssorchomene* that were collected at similar depth in the Kermadec Trench (Lacey et al., 2016). Adding these species would provide further evidence for a close similarity between the amphipod fauna of the Kermadec and Tonga trenches, as would be expected from their close proximity. However, the possibility of some differences in the amphipod assemblage between these two trenches was indicated by possibly two new species, and more detailed taxonomic work is required. Lacey et al. (2016) report possibly 17 undescribed species which await further study. Combining material across studies, ideally with the addition of molecular tools, will provide better insight into the regional trench faunas. A taxonomic review of these species, including DNA sequence analysis is underway and will be presented elsewhere (R. Peart et al., 2018, unpublished data).

Current biogeographic designations of hadal provinces separate the Bougainville–New Hebrides trenches from the Tonga–Kermadec trenches (Belyaev, 1989, and presented by Watling et al., 2013 as HD4 and HD5, respectively). However, Lacey et al. (2016) suggest that these hadal provinces be combined based on their shared amphipod communities which appear to be independent of the differences in overlying productivity regime and flux of POC to the seafloor. Our data support the contention of a single South West Pacific hadal province as proposed by Lacey et al. (2016) for the New Hebrides and Kermadec trenches, to now also include the Tonga Trench, due to the combined dominance of *H. dubia* and *B. schellenbergi* in all three trenches. Most of the species encountered at the trench edge site appear to be shared across these trenches. Our sites were located in the southern part of the Tonga Trench, between 250 and 290 km north of the Tonga sill that separates the Tonga and Kermadec trenches, however, despite this geographic proximity, the amphipod assemblage of the Tonga Trench shared a similar species richness with both the Kermadec and the New Hebrides Trench. Considering the north–south gradient of POC flux along the axis of the Tonga–Kermadec trenches, one alternative expectation might have been that amphipod assemblage composition of the Tonga Trench would reflect the similarity between the more oligotrophic Tonga Trench and the New Hebrides Trench, and be less like the mesotrophic Kermadec Trench to the south (at least toward its southern extent). However, this was not the case, and thus our data do not support a division of the hadal provinces into a separate Bougainville–New Hebrides province (HD4) and Tonga–Kermadec province (HD5) as originally proposed by Belyaev (1989) and repeated by Watling et al. (2013).

### Ontogenetic structure of *Hirondellea dubia*

The body size of the specimens was generally similar to that reported by Blankenship et al. (2006), although measurements were derived slightly differently (they used a proxy to determine total body length). The largest adult males (21.7 mm) and females (22.4 mm) measured here were slightly larger than previously reported (18.6 and 20.9 mm, respectively), and the smallest juvenile was larger at 4.8 mm compared to 2.8 mm previously reported.

The sex ratio of adult *H. dubia* in the present study showed a bias toward females. However, the observation at 6,250 m, where sex-indeterminate juveniles dominated, is based on a relatively small sample size, and deriving any significance from this ratio is probably unwise. However, the biased sex ratio determined from the population at the deepest depth in the Tonga Trench is based on a large sample size and can be considered for comparison with previous findings. In Blankenship et al. (2006) study in the Tonga Trench, *H. dubia* male-to-female sex ratios were 1:1 at a shallower depth of 7,000, while at 9,000–10,000 m, the proportion of females increased. However, beyond 10,000 m, the ratio of males to females return to 1:1 (Blankenship et al., 2006). The present study indicates that trend of an increasing proportion of females continues to the deepest extent of *H. dubia’*s known range (with a proportion of 0.64:0.36 for females and males, respectively, at 10,800 m). The change in sex ratio between the depths of 9,000 and 10,000 m observed by Blankenship et al. (2006) needs to be further examined, although it is likely that the change in ratio is associated with reproduction (Thurston, Petrillo & Della Croce, 2002; Ingram & Hessler, 1987).

While specimens examined in the present study come only from two sites at either extreme of the expected vertical distribution range of *H. dubia* in the Tonga Trench, the data analyses support previous findings of an ontogenetic shift with depth in the Tonga Trench. The abundance of *H. dubia* was much higher at the deepest site in the trench, where more advanced life stages dominated, compared to the shallower trench edge, where juveniles dominated the population. This difference is particularly apparent when considering late life stages where no fully mature life stages (M3, F4) were found at the shallower (6,250 m) depth. The proportion of younger life stages (F2, J, M2) was higher compared to older life stages (F3, F3a, F4, M3) at both depths, but the relative proportion of these was much smaller at the shallow site compared to the deep site. Additionally, where it was possible to compare, the size of animals of each life stage was larger at the deep trench axis site compared to the trench edge site, which was most pronounced in the juveniles. While the size range for the small number of subadults overlapped between depths, the largest juvenile at the 6,250 m site was smaller than the smallest juvenile at the 10,800 m site which indicates an ontogenetic shift between depths.

Ontogenetic stratification of trench amphipods has been previously observed in *H. dubia* (Blankenship et al., 2006), *H. gigas* (Eustace et al., 2013) and *B. schellenbergi* (Lacey et al., 2017), and various possible drivers have been proposed for these observations. Eustace et al. (2013) concluded that pressure alone cannot drive these observed trends but suggest an interaction of pressure and topography-influenced distribution of resources in terms of both quality and quantity. Lacey et al. (2017) argue that the distribution of juveniles does not relate directly to the distribution of food in a typical trench environment which funnels organic matter downslope toward the trench axis (citing Glud et al., 2013 and Ichino et al., 2015, also see Wenzhöfer et al., 2016) but instead conclude that the vertical distribution is driven by physiological and ecological factors. The former is clearly related to pressure where the biochemical processes at extreme depths result in low levels of enzyme activity which present metabolic limitations (Somero & Siebenaller, 1979; Somero, 1992; Siebenaller, 2010; Brown & Thatje, 2018). Therefore, juveniles may prefer shallower depths because it allows them to ingest and assimilate food more rapidly (Blankenship et al., 2006; Eustace et al., 2013). Whether this choice infers a reduced or increased predation pressure is contentious. Blankenship et al. (2006) argue that the upper depth limit of these amphipods reaches into the lower distribution limits of larger predators like fish or predatory decapods and that of other, larger, amphipods (also see Jamieson et al., 2009a, 2011; Jamieson, Solan & Fujii, 2009b). Lacey et al. (2017) suggest competitive interactions between *H. dubia* and *B. schellenbergi* at the upper depth limits of the trenches. However, they also suggest that the available space is larger at shallower depth compared to the axis of a trench. In addition, the total number of animals encountered on bait is much higher at the bottom of the trenches and raises the level of possible intraspecific competition and predation. We encountered about seven times as many amphipods in the traps in the Horizon Deep compared with the trench edge site, while Blankenship et al. (2006) captured >25 times as many animals in one trap at their deepest hadal site (800 animals) compared to a station very near our trench edge site (31 animals). And these numbers pale in comparison to numbers of animals caught in a single trap (17,800) at 8,723 m (Blankenship et al., 2006). Reduced intraspecific competition combined with the physiological advantages of a shallower depth appear to define the best strategy for survival in smaller amphipods, and the higher proportion of juveniles at the shallowest trench depth in the Tonga Trench appears to support this strategy. The mechanism by which juveniles are distributed to the shallower depths of their range is still uncertain (Blankenship et al., 2006; Lacey et al., 2017). Since amphipods are brooders, eggs are not passively dispersed, and it is still uncertain whether females or the juveniles actively migrate upward. As ovigerous females of a range of lysianassoid amphipods do not appear in baited traps, nothing is known about their vertical movement (Eustace et al., 2013; Kraft et al., 2013; Horton & Thurston, 2013; Lacey et al., 2017). But since food for scavenging amphipods is more plentiful at the trench axis (direct sediment measurements for both sites clearly indicate higher levels of organic activity and deposition rates for the trench axis compared to the edge, see Table 2) and considering the dominance of adults (and the presence of larger juveniles) of *H. dubia* at the axis, the most parsimonious explanation is that the animals migrate downslope with age. This migration would allow them to exploit the increased resource once they have grown large enough to effectively compete for food/avoid predation, as has also been inferred for *H. gigas* (Eustace et al., 2013) and *B. schellenbergi* (Lacey et al., 2017). With age, the animals might be able to overcome otherwise adverse physiological limitations imposed by higher pressure on younger developmental stages (sensu Smith, Brown & Thatje, 2015).

## Conclusions

Our study has expanded knowledge of the scavenging amphipod fauna of the Tonga Trench to 10 species, although some taxonomic and comparative work across the South West Pacific trenches is needed to solve species identities and describe up to 20 new species across all previous studies.

The ontogenetic vertical structuring of *H. dubia* in the Tonga Trench is confirmed by new data from the present study, and this distribution is likely driven by the distribution of food sources throughout the trench. There are strong incentives for juveniles to be distributed at shallower depths despite the presence of competing scavengers. The discovery that juveniles at the 6,250 m depth site were substantially smaller than at 10,800 m depth site supports the theory that juveniles migrate down the trench slope with increasing age. It seems likely that juveniles are distributed at shallower depths by brooding females, but there is still uncertainty around this speculation because no brooding females have yet been captured. Intense intraspecific competition for food is likely, and the results of the trap height analysis may demonstrate intraspecific exclusion, however, more research is needed in this area.

## Supplemental Information

10.7717/peerj.5994/supp-1 Supplemental Information 1 Morphometric data for Hirondellea dubia from Tonga Trench. NIWA catalog number, sex, stage and body measurements for both the ‘shallow’ (6,250 m) and ‘deep’ (10,800 m) site.

## Additional Information and Declarations

### Field Study Permissions

The following information was supplied relating to field study approvals (i.e., approving body and any reference numbers): Ministry of Foreign Affairs and Trade of the Government of Tonga, Nuku’alofa, 2 October 2013 (reference letter F.7/2/3).

### Data Availability

The following information was supplied regarding data availability: All the specimen records and the physical specimens are deposited at the NIWA Invertebrate Collection and its database in Wellington, New Zealand. The accession numbers of the amphipods are: 89965, 92761, 92762, 92763, 92764, 92765, 92766, 92767, 92768, 92769, 92770, 92771, 92772, 92773, 92774, 92775, 92776, 92777, 92778, 92779, 92780, 92796, 96621, 117111, 117115, 117116, 117117, 117118, 117119, 117120.
